# Snowfall and Carbon Monoxide Poisoning in Murree Pakistan

**DOI:** 10.12669/pjms.38.8.6936

**Published:** 2022

**Authors:** Muhammad Zain Sultan Meo, Muhammad Omair Sultan Meo, Anusha Sultan Meo

**Affiliations:** 1 Muhammad Zain Sultan Meo, College of Medicine, Alfaisal University, Riyadh, Saudi Arabia; 2 Muhammad Omair Sultan Meo, College of Medicine, Alfaisal University, Riyadh, Saudi Arabia; 3 Anusha Sultan Meo, College of Medicine, King Saud University, Riyadh, Saudi Arabia

Murree is a hill town 30 km to the northeast of Islamabad, Pakistan. The city is on an average altitude of 2,300 meters. On Friday, January 7, 2022, an estimated 10,000 people with over 1,000 vehicles went to enjoy the weekend amidst the breezy snowy weather in Murree. However, heavy snowfall ended up blocking the roads - to the point that some of the cars were packed bumper-to-bumper, with snow piling onto their roofs. About 22 people died inside their vehicles after the snow left them stranded inside. Causes of death were severe cold and carbon monoxide (CO) poisoning due to people leaving their car heaters on for too long. CO is an odourless, toxic gas formed due to partial combustion of hydrocarbons and fuel-burning, including wood, coal, natural gas, petroleum products, and petrol in motor vehicles.^1^ CO exposure during driving can occur due to defective exhaust systems, poor ventilation, and vehicle emissions.^2^ CO poisoning from petrol engine exhausts is a common accident and poisonings can be fatal. These unintentional CO poisonings witness an increase during the winter season.

**Fig.1:**
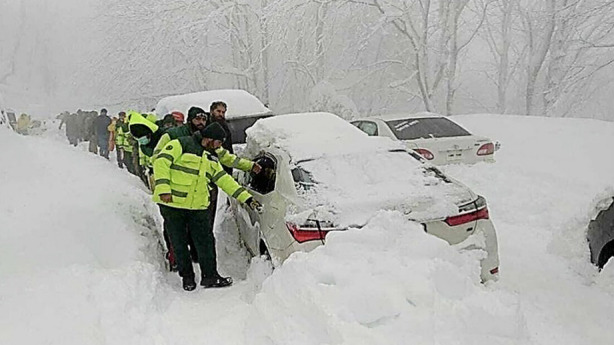
Motor vehicles situation due to Snowfall in Murree, Pakistan.

Environmental exposure to CO is less than 0.001% or 10ppm (ppm), and an active adult cigarette smoker is exposed to about 400-500 ppm of CO. This is in comparison to vehicle exhausts, which on the other hand can contain an overwhelming 100,000 ppm of CO as assessed inside a closed garage.^3^ Once inhaled, CO rapidly binds with haemoglobin (Hb) with a greater affinity than oxygen to form carboxyhemoglobin (CO-Hb). CO-Hb reduces the oxygen-carrying capacity and oxygen utilization. The CO-Hb level depends upon the concentration of the CO in the atmosphere and the exposure period.^3^,^4^ The CO-Hb level is less than 2% in non-smokers and 5% in smokers. A concentration over 9% is due to exogenous CO exposure.^5^ Hypoxia and toxicity can lead to cerebrovascular ischemia, myocardial infarction, and death. People with CO poisoning often overlook clinical symptoms such as headache, nausea, dizziness, shortness of breath, and confusion, and therefore this undetected exposure can be fatal.^1^ The global CO poisoning incidence and mortality is around 137 cases and 4.6 deaths per million populations.^6^

CO poisoning mainly occurs during the winter season; heating mechanisms can form CO gas in closed non-ventilated places, including motor vehicles. This is what happened in Murree, Pakistan, and cost the lives of 22 people who were found inside their cars. Since the cause is not a very well-known situation, people must be educated about this “silent killer”, and protective procedures should be taken to minimize CO poisoning-associated morbidity and mortality - regionally and globally.
